# Foot Mechanics Define Directional Changes in Curved-Path Walking: New Methods to Assess the Motor Skill of Walking

**DOI:** 10.1093/geroni/igab046.3700

**Published:** 2021-12-17

**Authors:** Haley Hicks, Anthony McBroom, Patrick Roscher, Jessie VanSwearingen, Kristin Lowry

**Affiliations:** 1 Broadlawns Medical Center, Des Moines, Iowa, United States; 2 Des Moines University, Des Moines, Iowa, United States; 3 Protokinetics, Inc, Havortown, Pennsylvania, United States; 4 School of Health and Rehabilitation Sciences, Pittsburgh, Pennsylvania, United States; 5 University of Pittsburgh, University of Pittsburgh, Pennsylvania, United States

## Abstract

Although it is essential to navigating the world, curved path walking is a challenge to mediolateral balance control. The focus of previous curved-path walking research was in spatiotemporal characteristics. We quantified the foot-ground interaction, center of pressure (COP) characteristics during non-linear (eg curved-path) walking important to understand the functional mechanics of directional changes for curved paths. We hypothesized the foot mechanics differ between older adults with better versus poorer curved-path walking (Figure of 8 Walk Test, F8W). Twenty-five older adults (mean age 71.8 ± 8.9 years) completed the F8W on an instrumented walkway (Protokinetics, LLC.) The derived metrics of the foot mechanics included medial/lateral movement of the COP for inside and outside steps, maximum medial and lateral COP excursions, and total medial/lateral COP range. Pearson correlations were used to examine relations F8W (time and steps) and COP metrics; ANOVAs were used to examine differences in COP metrics between older adults grouped by median-split of F8W time. Longer F8W time and more steps were related to lesser total COP range and outside foot lateral maximum excursion (r range -0.415 to -0.706, p<0.04). Older adults with stronger F8W performance compared to poorer F8W performance had larger outside foot total COP ranges (3.61cm vs 4.39cm, p=0.016) and greater lateral excursion (1.60cm vs 2.12cm, p=0,003). Foot-ground interactions offer new insights into control of curved path walking and methods for evaluating efficacy of interventions focused on improving walking skill in older adults.

